# Correlation Analysis of miR-1246 Expression in Saliva of Patients with Chronic Periodontitis and Periodontal Indexes, Inflammatory Cytokines, and Protease Molecules

**DOI:** 10.1155/2022/1949159

**Published:** 2022-07-30

**Authors:** Shuainan Wu, Zhaobao Li, Jianqi Wang, Lu Liu, Ying Pang

**Affiliations:** Stomatology Clinic of Cangzhou Central Hospital, Cangzhou, Hebei, China

## Abstract

**Objective:**

The study aimed to investigate the correlation of miR-1246 in saliva with periodontal indicators, inflammatory cytokines, and protease molecules in patients with chronic periodontitis.

**Methods:**

Thirty-five patients with chronic periodontitis were included as the chronic periodontitis group, and 35 healthy individuals were selected as the healthy control group during the same period. The miR-1246 levels, inflammatory cytokine interleukin (IL)-1*β*, IL-6, IL-17, tumor necrosis factor-*α* (TNF-*α*), matrix metalloproteinase (MMP-1), MMP-8, and MMP tissue inhibitor (TIMP-1) in saliva were determined, and periodontal indexes, including the plaque index (PLI), bleeding index (BI), periodontal probing depth (PD), and attachment loss (AL) were examined.

**Results:**

The salivary levels of miR-1246, IL-1*β*, IL-6, IL-17, TNF-*α*, MMP-1, MMP-8, and TIMP-1 and the periodontal indexes PLI, GI, PD, and AL in the chronic periodontitis group were significantly higher than those in the healthy control (*P* < 0.05). Salivary levels of miR-1246 in patients with chronic periodontitis were positively correlated with the levels of IL-1*β*, IL-6, IL-17, TNF-*α*, MMP-1, MMP-8, TIMP-1, PLI, GI, PD, and AL (*P* < 0.05).

**Conclusion:**

Abnormally elevated levels of miR-1246 in saliva of patients with chronic periodontitis correlate with levels of periodontal indices, inflammatory cytokines, and protease molecules.

## 1. Introduction

Chronic periodontitis is a chronic infectious disease of the gingiva and periodontal tissue caused by periodontal pathogens such as Porphyromonas gingivalis and Plasmodium intermedium. It can trigger host immune defense and inflammatory responses, leading to massive production of cytokines [1]. Inflammatory factors such as tumor necrosis factor-*α* (TNF-*α*), interleukin-1*β* interleukin (IL)-1*β*, IL-6, and IL-17 play an important role in the pathology of chronic periodontitis because they cause secondary damage to periodontal tissues thereby exacerbating the destruction of periodontal tissues [2]. It has been reported that TNF-*α*, IL-1*β*, IL-6, and IL-17 are highly expressed in periodontal tissues such as periodontal membranes of patients with chronic periodontitis as the expression level increases with the increase of inflammation [3]. MicroRNAs (miRNAs) are newly discovered endogenous nonprotein-coding single-stranded RNAs consisting of 21–24 nucleotides that regulate protein expression by degrading miRNAs or inhibiting their translation through complementary pairing with target gene mRNAs [4]. They are widely involved in the development of various diseases such as infectious diseases, immune diseases, cardiovascular diseases, and viral infections; i.e., changes in microRNA expression levels may indicate the presence of a disease [5].

The expression profiles of miRNAs in saliva are similar to those in tissues and plasma, and significant changes in specific miRNAs have been observed in saliva, diseased tissues, and blood [6]. There is increasing evidence that aberrant miRNA expression is closely associated with the pathogenesis of chronic inflammation. Thus, miRNAs in saliva show great potential as diagnostic and therapeutic targets for diseases. A recent study [7] revealed an important role of changes in miRNA expression levels, especially miR-1246, in the development of inflammation. However, changes in miR-1246 in chronic periodontitis in China and their significance have been rarely reported. Therefore, this study analyzed the correlation between miR-1246 levels in saliva of patients with chronic periodontitis and the levels of periodontal indicators, inflammatory cytokines, and protease-related molecules to elucidate the role of miR-1246 in the development of chronic periodontitis, as well as to provide a basis for exploring the pathogenesis of chronic periodontitis and finding new targets for future diagnosis and treatment.

## 2. Materials and Methods

### 2.1. General Data

Thirty-five patients with chronic periodontitis treated in our hospital from September 2020 to June 2021 were selected as the chronic periodontitis group, including 25 males and 10 females, aged 31 ∼ 67 years. Another 35 cases of healthy individuals under physical examination in our hospital during the same period were selected as the control group, including 23 males and 12 females, aged 32 ∼ 69 years. All patients met the diagnostic criteria for chronic periodontitis developed by the International Symposium on Periodontal Classification in 1999.

Inclusion criteria were as follows [8]: ① patients without oral or local inflammation, including tonsillitis and pharyngitis; ② patients without immune diseases, infectious diseases, or other systemic diseases; ③ patients without antibiotics or periodontal basic treatment in the last 3 months; ④ no pregnant or lactating women.

### 2.2. Apparatus and Reagents

The miRNA extraction kit, miRNA *c* DNA first-strand synthesis kit, miRNA fluorescence quantitative polymerase chain reaction (PCR) assay kit, IL-1*β*, IL-6, IL-17, tumor necrosis factor-*α* (TNF-*α*), and matrix metalloproteinase (MMP)-1, MMP-8, and MMP tissue inhibitory factor-1 (TIMP-1) ELISA kits were purchased from Beijing Convoy Century, Inc.

## 3. Methods

### 3.1. Determination of Periodontal Indexes

All subjects received an oral examination completed by the same physician, and the periodontal probing depth (PD) and clinical attachment loss (AL) were detected with the William periodontal probe; the plaque index (PLI) and the bleeding index (BI) were recorded.

### 3.2. Collection of Saliva Specimens

Samples were collected before treatment in the chronic periodontitis group and during physical examination in the healthy control group. In the morning, the mouth was wiped with a cotton swab dipped in 2% citric acid to collect about 1 ml of saliva in a 1.5 ml electrophysiology tube, followed by centrifugation at 3000 rpm for 15 min at 4°C to obtain the supernatant. The supernatant was divided into two portions and stored at −80°C.

### 3.3. Determination of miR-1246 Levels in Saliva

One portion of the supernatant was used for the determination of miR-1246 levels. The real-time fluorescence quantitative PCR was performed on an ABI 7500 fluorescence quantitative PCR instrument. The total PCR reaction system was 20 *μ*l: primer and probe 1 *μ*l mix (20×), TaqMan universal mix 10 *μ*l (2×), reverse transcription product cDNA 1.33 *μ*l, and nuclease-free water 7.67 *μ*l. The amplification conditions were as follows: 95°C for 10 min 1 cycle, 95°C for 15 s, and 60°C for 60 s 45 cycles. The number of cycles in each reaction system where the fluorescence signal reached the set threshold was the Ct value, and the expression level of miR-1246 was calculated using the 2^−△△Ct^ method. The delta-delta Ct method, also known as the 2^−△△Ct^ method, is a simple formula used to calculate the relative fold gene expression of a sample when performing real-time polymerase chain reaction (also known as qPCR). This method was devised in 2001 by Kenneth Livak and Thomas Schmittgen [9].

### 3.4. Determination of Inflammatory Cytokines and Protease-Related Molecules in Saliva

Another saliva sample was used for the determination of the levels of IL-1*β*, IL-6, IL-17, TNF-*α*, MMP-1, MMP-8, and TIMP-1 using the ELISA, and all assays were performed according to the kit's instructions.

### 3.5. Severity Grading of Periodontitis

Gingival inflammation and bleeding on probing, periodontal pocket depth ≤4 mm, radiographs showing alveolar bone resorption not exceeding 1/3 of the root length, and bad breath were considered mild according to severity grading. Gingival inflammation and bleeding on probing, also pus may be present, periodontal pocket depth ≤6 mm, X-ray showing alveolar bone resorption more than 1/3 of the root length but not more than 1/2 of the root length, and the teeth having mild loosening were considered moderate according to severity grading. More pronounced inflammation or periodontal abscess, periodontal pocket >6 mm, X-ray showing alveolar bone resorption more than 1/2 of the root length, and the tooth being mostly loose were considered severe according to severity grading [10, 11].

### 3.6. Statistical Analyses

SPSS 23.0 software was used for data processing and statistical analysis. The measurement data were all continuous variables conforming to the normal distribution, expressed as the mean ± standard error, and the *t*-test was used for comparison between groups. The count data were expressed as percentages, and the chi-square test was used for comparison between groups. Pearson correlation was used to analyze the correlation between the indicators. A difference was considered statistically significant at *P* < 0.05.

## 4. Results

### 4.1. Comparison of General Data

There was no statistically significant difference between the two groups of patients in terms of general data such as age and gender (*P* < 0.05) (see Table 1).

### 4.2. Comparison of miR-1246 Levels

The miR-1246 level of the chronic periodontitis group was 2.13 ± 0.41, which was significantly higher than that of 0.92 ± 0.11 in the healthy control group (*P* < 0.05) (see Table 2).

### 4.3. Comparison of Periodontal Indexes

Compared with the healthy control group, the chronic periodontitis group had significantly higher levels of PLI, BI, PD, and AL (*P* < 0.05), as shown in Table 3.

### 4.4. Comparison of Salivary Levels of Inflammatory Cytokines

Significantly higher levels of IL-1*β*, IL-6, IL-17, and TNF-*α* were found in saliva of the chronic periodontitis group compared with the healthy control group (*P* < 0.05), as shown in Table 4.

### 4.5. Comparison of Protease-Related Molecule Levels in Saliva

The levels of MMP-1, MMP-8, and TIMP-1 in saliva were markedly higher in the chronic periodontitis group than those in the healthy control group (*P* < 0.05), as shown in Table 5.

### 4.6. Correlation of miR-1246 Levels with Inflammatory Cytokines and Protease Molecules in Saliva of Patients with Chronic Periodontitis

**Figure 1:**
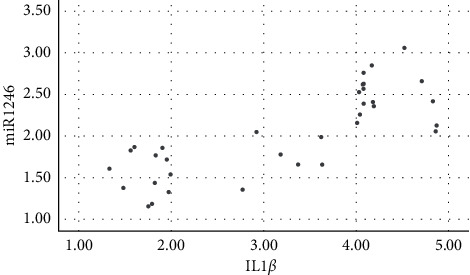
Positive correlation between miR-124 and IL-1*β*.

**Figure 2:**
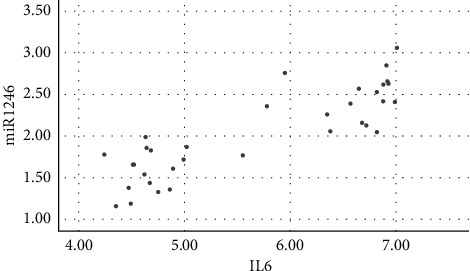
Positive correlation between miR-124 and IL-6.

**Figure 3:**
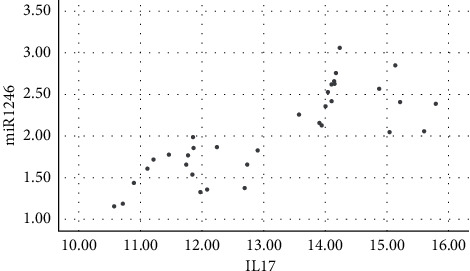
Positive correlation between miR-124 and IL-7.

**Figure 4:**
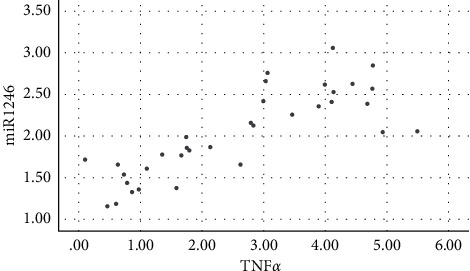
Positive correlation between miR-124 and TNF-*α.*

**Figure 5:**
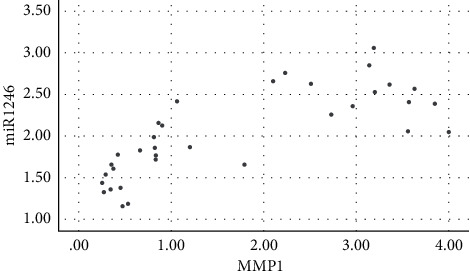
Positive correlation between miR-124 and MMP-1.

**Figure 6:**
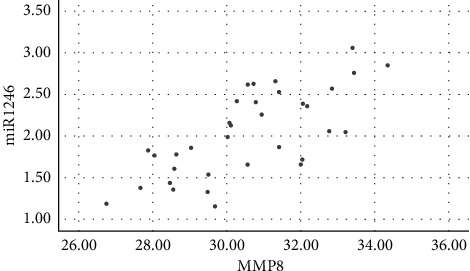
Positive correlation between miR-124 and MMP-8.

**Figure 7:**
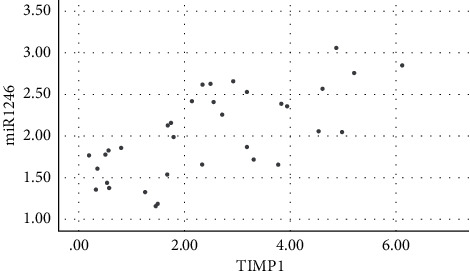
Positive correlation between miR-124 and TIMP-1.

Salivary levels of miR-1246 in patients with chronic periodontitis were positively correlated with the levels of IL-1*β*, IL-6, IL-17, TNF-*α*, MMP-1, MMP-8, and TIMP-1 (*P* < 0.05), as shown in figures 1–7.

## 5. Discussion

Chronic periodontitis is an inflammatory and destructive disease of periodontal supporting tissues, characterized by alveolar bone resorption and gingival recession and has become a common chronic disease that endangers human oral health [12]. It is a chronic or acute recurrent progressive inflammatory disease caused by plaque, and its products act on periodontal tissues and activate the host's inflammatory immune response [13]. The inflammation they cause extends to deeper tissues and leads to the destruction of periodontal tissues [14]. Any factor that can aggravate plaque retention, such as tartar, poor restorations, food impaction (plugged teeth), misalignment of teeth, and abnormal anatomical morphology, can become a local promoter of periodontitis, aggravating and accelerating its progression [15]. Periodontitis is a multifactorial disease, and certain systemic diseases such as diabetes mellitus can also negatively affect periodontitis [16]. No systemic disease or dysfunction is thought to directly contribute to the development of chronic periodontitis [17]. In addition, certain environmental and behavioral factors such as smoking and mental stress may be risk factors. In recent years, it has been found that genetic background may also be associated with chronic periodontitis [18].

miRNA, as an endogenous noncoding small molecule, is involved in the regulation of post-transcriptional gene expression and plays a role in the pathogenesis of periodontitis [19]. Previous studies have found that aberrant miR-1246 expression in inflammatory diseases could show great potential as an important biomarker for monitoring inflammatory disease status because miR-1246 is involved in inflammatory factor signaling and regulation of immune cell differentiation and immune function [20]. In recent years, the role of host inflammatory immune response in periodontal disease progression has been well studied and recognized. Periodontal cell populations are heterogeneous multifunctional cell populations containing stem cells, fibroblasts, osteoblasts, dentin-forming osteoclasts, Malassezia epithelial remnants, immune cells, undifferentiated mesenchymal cells, and dentin-forming cell vesicles [21]. During inflammatory episodes, host immune cells fight pathogenic microorganisms, kill bacteria, and participate in the immune response [22]. miR-1246 regulates the activation of monocytes, macrophages, and neutrophils in T-cell-mediated and B-cell-mediated immune responses [23]. Adeodato et al. (2020) found in a miR-1246 knockout mouse hair growth center model that B-cell production with a corresponding decrease in interleukin 2 and interferon *γ* production have loss of the ability to produce high-affinity IgG1 antibodies, TNF-*α*, and lymphotoxin [24].

Activation of the inflammatory response in plaque-stimulated periodontal tissues is the underlying pathological change in chronic periodontitis, and periodontal tissues release large amounts of inflammatory factors such as IL-1*β*, IL-6, IL-17, and TNF-*α* [25]. The elevated levels of these inflammatory cytokines in the gingival sulcus fluid of patients with periodontitis have been demonstrated, and the results of the analysis in the present study are consistent with previous studies [26]. Furthermore, in the present study, the levels of miR-1246 in saliva of patients with chronic periodontitis were positively correlated with the levels of IL-1*β*, IL-6, IL-17, and TNF-*α*, indicating a correlation between the two. With the release of inflammatory factors and activation of the inflammatory response, miR-1246 levels increased. Activation of the inflammatory response in periodontal tissues can lead to local infiltration of multiple inflammatory cells, release of proteases, and inhibitory molecules such as MMP-1, MMP-8, and TIMP-1 that mediate periodontal tissue hydrolysis and destruction [10, 27]. The results of this study also showed a positive correlation between MMP-1, MMP-8, TIMP-1 levels, and miR-1246 levels in the chronic periodontitis group, suggesting that increased miR-1246 expression may be associated with the destruction of periodontal tissues by proteases in chronic periodontitis.

Imaging evaluation can provide information about the severity of periodontitis based on its findings of probing depth, attachment level, bleeding detection, and alveolar bone loss but is relatively expensive and unable to detect disease activity [28, 29]. Many herbs are very effective in preventing chronic diseases, and herbs can be effective in the prevention, treatment, or prognosis of chronic periodontitis [30, 31]. In Chinese medicine, the identification of periodontitis is mainly related to the dysfunction of the internal organs, with the incandescence of stomach heat and deficiency of kidney qi being the most common [32]. However, when periodontitis occurs, it cannot be cured radically if it is treated only by TCM, so a combination of western medicine is also needed [33]. In clinical practice, gingivitis can gradually and insidiously transition into periodontitis, so early detection and diagnosis of periodontitis are important because the consequences of periodontitis are far more serious than gingivitis [34, 35]. Therefore, it is important to find efficient diagnostic indicators and new therapeutic targets, which is the significance of the study we conducted.

## 6. Conclusion

In conclusion, abnormally elevated levels of miR-1246 in saliva of patients with chronic periodontitis correlate with levels of periodontal indices, inflammatory cytokines, and protease molecules, which indicate their involvement in the development of chronic periodontitis. However, due to the small sample size and the short follow-up period, future studies with expanded samples and longer follow-up periods are required.

## Figures and Tables

**Table 1:** Comparison of general data between the two groups.

Groups	*n*	Gender		Age (year)	Hypertension	Smoking	Drinking
		Male	Female				
Health control group	35	25	10	57.87 ± 9.26	13	16	13
Chronic periodontitis group	35	23	12	57.42 ± 7.75	17	19	15
*χ* ^2^ */t*		0.032		0.824	0.142	0.342	0.216
*P*		0.825		0.126	0.726	0.861	0.798

**Table 2:** Comparison of miR-1246 levels between the two groups.

Groups	*n*	miR-1246 level	*t*	*P*
Health control group	35	0.92 ± 0.11	14.367	≤0.001
Chronic periodontitis group	35	2.13 ± 0.41		

**Table 3:** Comparison of periodontal indexes between the two groups.

Groups	*n*	PLI	BI	PD (mm)	AL (mm)
Health control group	35	0.89 ± 0.12	1.09 ± 0.22	1.82 ± 0.47	0.73 ± 0.15
Chronic periodontitis group	35	3.13 ± 0.51	3.47 ± 0.79	6.61 ± 0.88	7.65 ± 1.17
*t*		22.872	18.472	29.026	39.461
*P*		≤0.001	≤0.001	≤0.001	≤0.001

**Table 4:** Comparison of salivary levels of inflammatory cytokines between the two groups.

Groups	*n*	IL-1*β* (pg/ML)	IL-6 (pg/ML)	IL-17 (pg/ML)	TNF-*α* (pg/ML)
Health control group	35	1.89 ± 0.37	3.01 ± 0.59	7.64 ± 0.91	0.93 ± 0.12
Chronic periodontitis group	35	3.45 ± 0.63	5.76 ± 0.82	12.52 ± 3.01	2.38 ± 0.51
*t*		12.753	16.842	10.486	16.771
*P*		≤0.001	≤0.001	≤0.001	≤0.001

**Table 5:** Comparison of protease-related molecule levels in saliva of the two groups.

Groups	*n*	MMP-1	MMP-8	TIMP-1
Health control group	35	1.68 ± 0.31	12.41 ± 2.45	3.08 ± 0.59
Chronic periodontitis group	35	3.82 ± 0.79	33.07 ± 7.13	4.18 ± 0.82
*t*		15.862	18.371	7.307
*P*		≤0.001	≤0.001	≤0.001

## Data Availability

The datasets used and analyzed during the current study are available from the corresponding author on reasonable request.

## Abbreviations

(PLI): Plaque index
(BI): Bleeding index
(PD): Periodontal probing depth
(AL): Attachment loss
(PCR): Polymerase chain reaction.
